# Determination of oxygen extraction fraction using magnetic resonance imaging in canine models with internal carotid artery occlusion

**DOI:** 10.1038/srep30332

**Published:** 2016-07-22

**Authors:** Fei-Yan Chang, Jiang-Xi Xiao, Sheng Xie, Lei Yu, Zhen-Xia Zhang, Wu Wang, Jie Luo, Zhong-Ping Zhang, Hua Guo

**Affiliations:** 1 Department of Radiology, China-Japan Friendship Hospital, BeiJing, 100029, China; 2 Department of Radiology, Peking University First Hospital, BeiJing, 100034, China; 3 Department of Pathology, China-Japan Friendship Hospital, BeiJing, 100029, China; 4 GE Healthcare Research, Beijing, 100176, China; 5 Center for Biomedical Imaging Research, Department of Biomedical Engineering, Tsinghua University, Beijing, 100084, China

## Abstract

Perfusion of the penumbra tissue below the flow threshold for functional disturbance but above that for the maintenance of morphological integrity is the target for therapy in acute ischaemic stroke. The measurement of the oxygen extraction fraction (OEF) may provide a direct assessment of tissue viability, so that irreversible tissue damage and penumbra can be reliably identified. By using an asymmetric spin echo single-shot echo planar imaging (ASE-SSEPI) sequence, the quantitative OEF was obtained in the ischaemic brain tissues of canine models with internal carotid artery occlusion. TTC staining, which delineated the regions of infarct and penumbra, was used for defining the corresponding regions on OEF maps. The threshold of the OEF to discriminate the infarct cores and penumbral tissues was then determined according to the OEF values at different times. With repeated-measures ANOVA, the OEF of the infarcted regions was found to be time dependent. An OEF greater than 0.48 best predicted cortical infarction at 1.5 hr, with an area under the receiving operating characteristic curve of 0.968, a sensitivity of 97.5%, and a specificity of 92.5%. Our results may be helpful in the evaluation of tissue viability during stroke events.

The occlusion of a cerebral artery can lead to the reduction of oxygen and substrate supply to brain tissues and may further result in brain ischaemia and eventual infarction. Active treatment of acute ischaemic stroke can be successful only as long as tissue in the area of ischaemic compromise is still viable; therefore, the identification of the area of irreversible damage, and its distinction from the penumbral zone, may improve the estimation of the potential efficacy of various therapeutic strategies. The oxygen extraction fraction (OEF) has been recognised as an accurate and specific marker for tissue viability and a predictor of misery perfusion in patients with chronic ischaemic diseases^1^,^2^,^3^,^4^. Imaging tissue oxygen metabolism may provide a more direct assessment of tissue viability^5^. Thus far, positron emission tomography (PET) has been the gold-standard technique to measure cerebral oxygen metabolism quantitatively and *in vivo*^6^,^7^,^8^. However, owing to the high cost and radiation of the injected tracers, the use of PET in characterising cerebral hemodynamic status is restricted. Using the magnetic resonance imaging (MRI) approach to estimate the OEF is now feasible with the advent of fast imaging techniques. On the biophysical basis of BOLD contrast mechanisms, the concentrations of deoxyhaemoglobin in the cerebral capillaries and veins as an indicator of the OEF can be visualised by T2*-based imaging^9^. An and colleagues have reported an innovative MR technique to estimate the OEF and cerebral metabolic rate of oxygen (CMRO_2_) of the human brain under normal and hypercapnia conditions using either a multi-echo gradient echo/spin echo (MEGESE) or an asymmetric spin echo (ASE) EPI sequence^10^,^11^,^12^. The OEF values measured by MRI are in good agreement with those reported in PET literatures^13^,^14^. Furthermore, high linear correlations have been demonstrated between the MR-measured cerebral oxygen saturation and blood gas oximetry measurements in the jugular veins of rats under global cerebral oxygenation alterations^15^. The imaging OEF using MRI has also been applied clinically in patients with cerebrovascular disease and patients with MELAS^16^,^17^.

The concept of penumbra in ischaemia, based on animal experiments, is defined as the potentially reversible functional disturbances that can be observed when blood flow decreases beyond a critical threshold^18^. However, the detailed relationship between the OEF derived from MR imaging and pathophysiological information in the ischaemic brain has not been investigated. Likewise, the threshold of OEF for distinguishing the penumbra or infarct regions from normal cortical tissues in the ischaemic brain still needs to be established.

To this end, the OEF values of canine models with carotid artery occlusion were measured using MRI in this study. To understand the relationship between the OEF value and pathophysiological information in ischaemic regions, OEF maps were compared with matched pathological specimen slices. Thus, the threshold of the OEF between the penumbra and infarct regions was defined.

## Materials and Methods

### Animal procedures and experimental protocol

All of the animal protocols were approved by the Institutional Animal Care and Use Animal Ethics Committee of Peking University First Hospital (Grant No. J201316). This study was carried out in strict accordance with the approved guidelines. Eleven mongrel dogs of both sexes weighing approximately 13 to 15 kilograms were included in this study. A dog was selected randomly as the control. The control dog was not operated upon to generate the ischaemic model but was sacrificed for pathological observation on the brain. All of the animals were anaesthetised with an intravenous injection of 2.5% pentobarbital (1 ml/kg), and anaesthesia was maintained with pentobarbital at 3 mg/kg per hour. The baseline MRI scans of the normal state (baseline) were performed on all canines. Next, blockade of the blood flow of the bilateral internal carotid arteries (ICAs) was induced by bilateral ICA ligation of all canines except the control one. Further embolisation was applied by the injection of comminuted sutures into both ICAs through an endovascular catheter. Additionally, a Headhunter 5 F catheter was inserted into the superior sagittal sinuses for continuous monitoring and sampling of blood for the analysis of blood gases. After the surgery, MRI examinations and blood gas analysis were performed every 30 minutes to monitor the perfusion and oxygenation of the brain. The last examination was at 2 hrs after surgery.

At the end of each experiment, the dogs were euthanised by intravenous injection of potassium chloride under general anaesthesia. Next, all of the canine brains were harvested immediately through craniotomy, frozen at −20 °C for 30 min, and sliced coronally at 2- to 3-mm intervals. All of the coronal brain sections were placed in 2% 2,3,5-triphenyltetrazolium chloride (TTC) for 45 minutes at 37 °C and were photographed.

### Magnetic resonance imaging

All MRI scannings were performed using a 3.0 T MRI scanner (GE Discovery MR 750; General Electric Company, Milwaukee, Wisconsin, USA) with an 8-channel head coil. A cotton pad was used to maintain the thermal stability of the dogs, and oxygen was supplied with a nasal oxygen catheter connected to an oxygen bag. The head of each canine was fixed in a hard box, and the axial plane of the brain was kept perpendicular to the main magnetic field. Scanning sequences included coronal T_2_-weighted Imaging, diffusion-weighted imaging (DWI), asymmetric spin echo single-shot echo planar imaging (ASE-SSEPI), 3D pseudo continuous arterial spin labelling (3D PCASL) and MR angiography (MRA). MRA was used to identify the loss of blood flow in the vessels. 3D PCASL, using intravascular water as an endogenous contrast agent, can measure the cerebral blood flow (CBF) quantitatively and accurately. On the basis of previous studies, an ASE single-shot EPI sequence was adopted to obtain estimates of the OEF with a different time shift τ, which represents the time shift of the 180° pulse relative to the original position. The parameters for ASE-SSEPI were as follows: TR = 4000 ms, TE = 77.1 ms, flip angle = 90°, slice thickness/gaps = 5 mm/1 mm, in-plane matrix = 64 × 64, NEX = 2, FOV = 12 cm × 12 cm, 4 echoes with τ = 0, and 19 echoes with τ = 1,2,3,…,19 ms, 23 echoes in total. Data were collected for 6 slices with the centre of the FOV set at the basal ganglia and thalamus. For the repeatability test, the baseline scanning was repeated twice. After the surgery, the above sequences were repeated every 30 minutes after the onset of occlusion.

### Parameter estimation

The OEF values were estimated using previously described techniques^10^. The program was embedded in a GE post-processing workstation (GE Healthcare, ADW4.5). ASE-SSEPI images were transported to the workstation, and OEF maps were calculated. In addition, images of the 3D PCASL sequence were directly processed on the GE console, and CBF maps were generated.

### Pathological assessment

The canine cortical slices stained with TTC were evaluated by an experienced pathologist. In ischaemic tissues, the lack of TTC staining is considered ‘infarcted’ and is defined as “cores”, and viable tissues are stained red^19^. The penumbra region is characterised by strong staining but with swelling of the cortex and indistinctness of the corticomedullary border. The locations of the core and penumbra were recorded for each slice corresponding to the MRI scanning sections. Subsequently, the slices with basal ganglia and thalamus were stained with haematoxylin and eosin (HE) to confirm the infarcted and viable tissue.

### Data analysis

**Figure 1:**
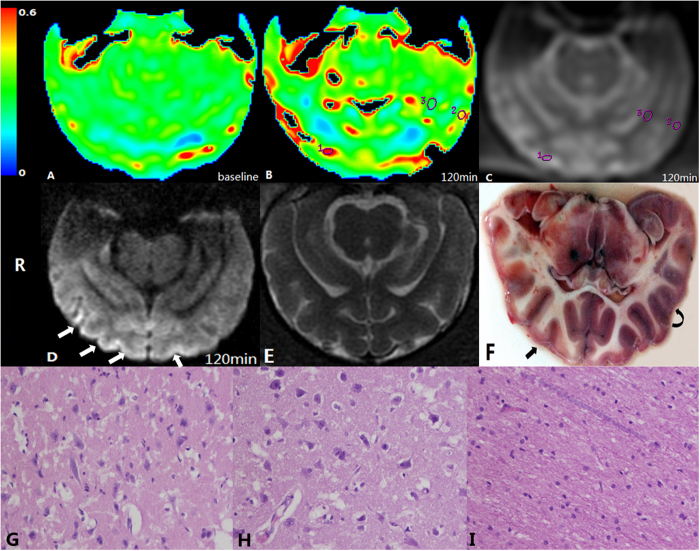
Illustration of ROI placement. (**A**) OEF at baseline, (**B**) OEF map at 2 hrs after embolisation. ROIs were placed in a core (ROI 1), an ischaemic penumbra (ROI 2), and normal white matter (ROI 3). (**C**) Raw image of the ASE sequence, which is used to locate the ROIs. (**D**) DWI image at the same slice as the OEF map. The cortices of the right hemisphere and medial cortex of the left hemisphere showed a high signal (white arrow). E: T_2_WI image of the same slice as OEF map and DWI image. (**F**) TTC staining slice corresponding to the DWI image and OEF map. Infarct lesions in cortices of the right hemisphere are depicted (straight arrow) that were whitened and consistent with a high signal on DWI imaging. Some cortices in the left hemisphere were swollen but with normal colour, defined as ischaemic penumbra (curved arrow). (**G–I**) Microscopic views of the core (ROI 1), penumbra (ROI 2), and white matter area (ROI 3) (haematoxylin and eosin staining, ×400). Marked neuronal degeneration is shown in the core (**G**) whereas cell oedema is predominant in the penumbra region (**H**). Normal white matter depicts normal neuronal cells without nuclear karyolysis and neuronal cell loss (**I**).

In MR images, only the two brain sections containing basal ganglia and thalamus were analysed. Sections in the anterior and posterior proximity were excluded because of extensive susceptibility artefacts from the bone-air interface. Even in the middle sections, there were some regions just below the cranial bone showing a high signal on the baseline OEF map. These regions were not included in the analysis. Moreover, some brain regions with low signal-to-noise ratios resulting from artefacts, or with data unsuitable for analytical processing, were also excluded from further analysis. The ROIs of the infarct prescribed on the OEF maps were maintained to be consistent with both cores defined on TTC staining and cortical regions with high signal on DWI images. DWI detects cytotoxic oedema, owing to the restricted motion of the water molecules trapped in the cell^20^. The penumbra regions were first determined on the TTC staining sections. Next, the ROIs of the penumbra regions were prescribed on the corresponding brain regions with hypoperfusion but without restricted diffusion in MRI images. In addition, 40 ROIs of the deep white matter were prescribed, which were presumed to represent relatively normal brain tissues. The sizes of ROIs ranged from 4 to 14 mm^2^. On the corresponding OEF maps, values of the baseline and follow-up scannings at 30 min, 60 min, 90 min and 120 min after surgery were obtained for each ROI. For illustration, the detailed determination of the infarct cores, penumbra and normal white matter regions of a typical dog are presented in Fig. 1.

On CBF maps, two circular ROIs of the size approximately 200 mm^2^ were placed in the bilateral hemispheres in every cerebral section to compare CBF values at baseline and at 2 hrs after embolisation. The same ROIs were prescribed on OEF maps as well, and the OEF values of both hemispheres at baseline and at 2 hrs after embolisation were compared.

### Statistical analysis

The OEF and CBF values were expressed as the means ± standard deviation. Repeatability of the OEF measurements was evaluated by calculating the intra-class correlation coefficients (ICCs), standard deviation (SD) and coefficient of reproducibility (CR) of the two repeated baseline OEF measurements. To study the temporal variation of the OEF in the infarct core, penumbra and normal white matter, repeated-measures analysis of variance (ANOVA) was applied in which the group was treated as a main factor, and the time interval was treated as a repeated factor. In addition, changes of OEF after ICA occlusion were compared among the ROIs of the infarct core, penumbra and normal white matter at different time points by using Kruskal-Wallis test. Receiver operating characteristic (ROC) curve analyses were performed to assess whether the OEF values could predict infarction at 0.5–2 hrs. For the OEF of each time point, ROC analysis was used to estimate the area under the ROC curve, the criterion associated with the highest sensitivity and specificity, and the sensitivity and specificity associated with the selected criterion. All of the reported *P* values were calculated with two-sided tests, and *P* values < 0.05 were considered to be statistically significant.

## Results

First, the measurements of the OEF using MRI technique were proven to be repeatable in our study, with an ICC value of 0.962, a CR value of 1.665% and a mean SD of 0.005.

Second, the results of blood gas analysis indicated that occlusion of the ICA led to statistically significant changes in the blood oxygen saturation of the superior sagittal sinus, decreasing from 88.45% preoperatively to 59.60% postoperatively (*P* < 0.05).

**Figure 2:**
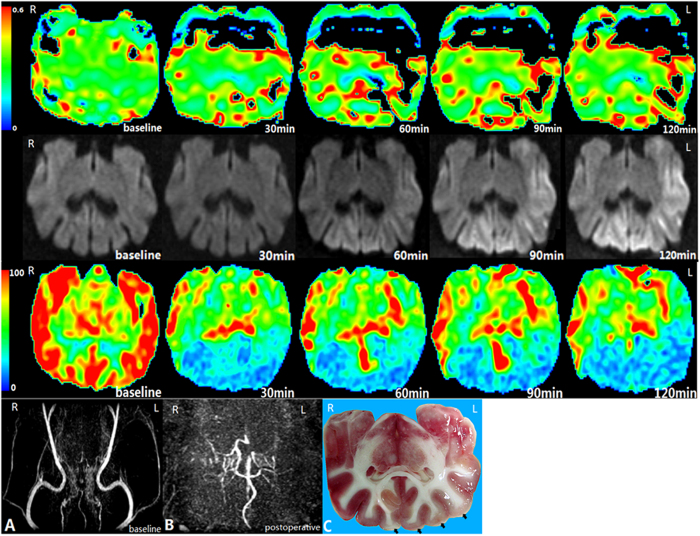
Bilateral occlusion of ICAs in canine 9. The first row, second row, and third row: OEF, DWI, and CBF images at baseline, 30 min, 60 min, 90 min, and 120 min after the bilateral occlusion of ICAs. The fourth row: MRA image at baseline; B MRA image after occlusion; C TTC staining slice. The bilateral cortices (black arrows) are shown to be histologically infarcted.

The results of MRA demonstrated the disappearance of blood flow in the ICA subjected to embolisation, as shown in Fig. 2.

The CBF maps revealed a statistically significant reduction of blood flow in the hemispheres postoperatively (Fig. 2). The CBF values decreased from 59.56 ± 25.98 ml/(min.100 g) at baseline to 34.24 ± 12.59 ml/(min.100 g) at 2 hrs after occlusion (*T* = 8.75, *P* < 0.01). Ischaemic brains showed a significant elevation in the OEF after embolisation, from 0.30 ± 0.01 at baseline to 0.36 ± 0.06 at 2 hrs after embolisation (*T* = −9.11, *P* < 0.01).

**Figure 3:**
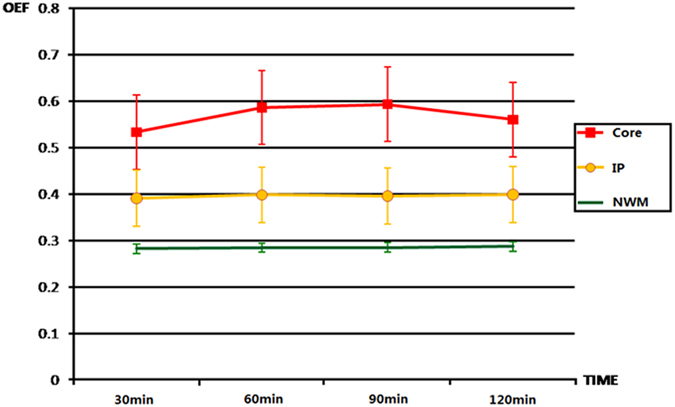
Shows dynamic changes in the postoperative OEF in the ROIs of the infarct core, ischaemic penumbra (IP), and normal white matter (NWM). The time course of changes in the OEF was comparable in the IP and NWM, while the profile of the OEF changes in the cores was not similar to them.

From our observations of TTC staining, CBF and DWI images, we found that more of the perfusion-diffusion mismatches corresponded to regions with oligaemia than with penumbra. According to our settings, we identified 40 foci of infarct cores and 32 foci of penumbra regions from all of the ischaemic brains for further analysis. Most of these regions were located in the gyrus coronalis, gyrus postcruciatus and gyrus ectosylvius. Elevation of the OEF was found in these regions on OEF maps, a finding that was in agreement with ischaemic foci on TTC staining sections. Accordingly, the mean OEF values of the infarct and penumbra were determined as shown in Table 1. The post-hoc comparisons indicated significant differences among the infarct cores, penumbra regions and normal white matter areas (Bonferroni corrected *P* = 0.001). The OEF values in the infarct cores were significantly higher than those in all other ROIs at each detected time point. Penumbra regions showed moderately but significantly higher OEF values than normal white matter. Furthermore, a significant “time × group” interaction of the OEF was observed (*P* = 0.002), thus suggesting different temporal variations of the OEF among different groups. Figure 3 shows the dynamic variation of the postoperative OEF in the ROIs of the infarct core, penumbra, and normal white matter. In infarct cores, the OEF values were found to increase with infarction time, except for the last time point. In contrast, the OEF values in the penumbra regions and normal white matter areas did not demonstrate any significant change with time. The haemodynamic changes in the CBF and OEF in a dog with embolisation of the bilateral ICA are shown in Fig. 2. The CBF values in the bilateral hemispheres were significantly reduced compared with those of the baseline. DWI images demonstrated restricted diffusion in the grey matter of the bilateral hemispheres, indicating cytotoxic oedema. From Table 2, it was indicated that there were significant differences in changes of OEF among the infarct core, penumbra and normal white matter after occlusion at different time points.

**Figure 4:**
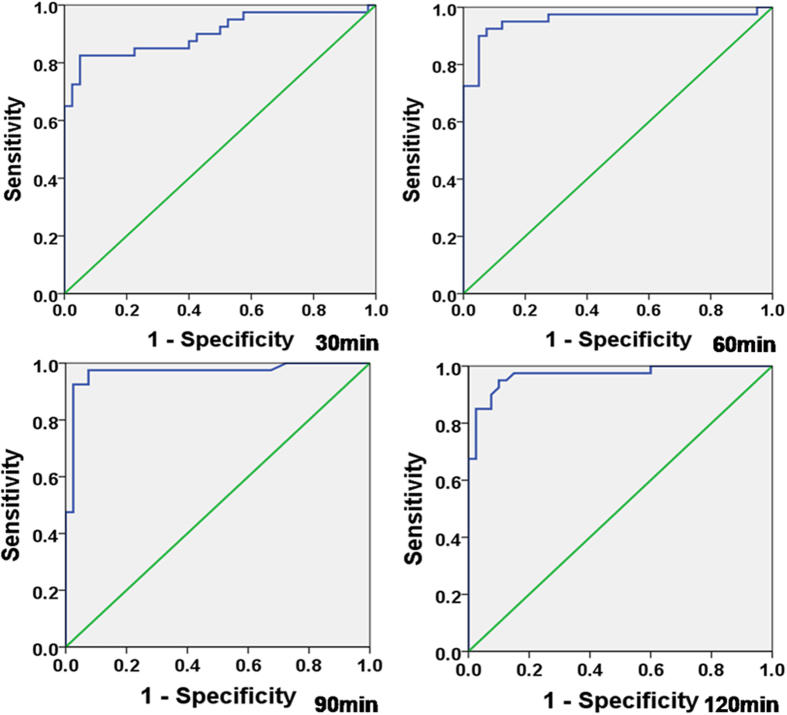
ROC curves analysing the OEF to discriminate between penumbra and infarction for each time point.

The areas under the ROC curves for the OEF were 0.902, 0.956, 0.968, and 0.969 at 0.5 hr, 1 hr, 1.5 hr, and 2 hrs, respectively. (Table 3, Fig. 4) The parameter most predictive of infarction was the OEF at 1.5 hr, with a sensitivity of 97.5% and a specificity of 92.5%, when the cut-off value was set as 0.48. Figure 4 depicts ROC curves analysing the ability of the OEF at each time point to discriminate between the penumbra and infarction.

## Discussion

In the present study, using experimental ischaemic canine models, the OEF values were estimated with MRI. We provide the first report of the threshold of the OEF values of the penumbra and infarct regions being identified through a direct comparison with the pathological specimen. The determination of the MR OEF on the basis of results from pathological assessment might aid in the interpretation of the respective findings and in the assessment of the tissue viability for predicting tissue outcome.

CBF, OEF, and CMRO_2_ can all be used as indices reflecting alterations of cerebral haemodynamics and metabolism during cerebral ischaemic insults. Theoretically, after the onset of the cerebral ischaemia, the OEF value is elevated to compensate for the reduced CBF and to maintain a stable CMRO_2_ to preserve neuronal function or cellular integrity^6^,^21^,^22^,^23^. Among them, the CBF is highly variable across the brain regions and can predict irreversible cortical damage in a time-dependent manner^24^. However, there are several methodological limitations, especially regarding perfusion techniques, and data evaluations are not truly quantitative and vary among centres^25^. The reduction of CMRO_2_ may be a more specific marker than that of the OEF or CBF in delineating tissue viability^7^. An *et al*. have demonstrated dynamic alteration of cerebral oxygen metabolism during acute ischaemia in rats with the MRI-measured cerebral oxygen metabolic index. However, this index was calculated as the product of the OEF and CBF in their study, thus suggesting that it can theoretically be compromised by intrinsic errors from the estimation of both CBF and OEF. Relative to the MR-derived CMRO_2_, the MR OEF measurement seems to be a more applicable and reliable technique. Our experiments revealed good repeatability of MRI-based OEF measurements among scans. Additionally, OEF is a sensitive surrogate to predict brain tissues at risk^16^,^26^. In our study, comparison of the OEF map and TTC staining slices showed good consistency between the brain regions with elevated OEF and ischaemic foci on the slices (Fig. 2).

During the ischaemic insult, cortices with higher OEF values tend to exhibit increased morphological destruction. In this study, the threshold of the OEF value for the infarction was 0.48 at 1.5 hr and 0.45 at 2 hrs after the onset, with high sensitivity and specificity. Using PET, the quantitative assessment of the OEF values of the ischaemic tissues was extremely difficult. The threshold of the PET-detected OEF values of morphological damage and the upper limit of the penumbra regions have been reported to vary among different studies^18^. As a result, there is no accepted threshold. Absolute hemispheric OEF values beyond the upper 95% confidence limits defined in healthy volunteers are usually considered to be abnormal^27^. According to our observations, brain regions with elevated OEF values consist of penumbral tissues and tissues undergoing final infarction. With the threshold, it is possible to distinguish infarct cores from penumbral tissues. However, some authors have suggested that the OEF may exhibit biphasic behaviour during ischaemia, thus making it difficult to define a single viability threshold^8^,^21^. Moreover, the threshold of the infarct cores was affected by the time of determination after the vascular attack in this study, showing a mainly progressive increase with a subsequent decrease. As shown in Fig. 3, the OEF in infarct cores at 2 hrs after the attack unexpectedly decreased compared with the earlier time point. A previous study has reported that the brain regions in which the OEF is initially elevated are consistently associated with an eventual infarction that in turn causes a secondary decrease in the OEF to abnormally low values^28^. The evolution of the OEF in this study may be affected by the local neuronal loss and/or spontaneous reperfusion occurring in the cortex. This phenomenon was remarkably prominent in the basal nuclei. It is possible that, in the deep MCA territory, in the future infarct area, there was a severe and progressive metabolic deterioration driving the OEF back towards normal values. This interpretation has been proposed in baboon studies^8^. An *et al*. have also reported that severe ischaemic tissues might evolve very quickly and reach a low oxygen metabolic state early after MCA occlusion^15^.

Determination of the penumbra is important in the clinical management of an ischaemic event. Thus far, magnetic resonance diffusion perfusion mismatch has been used to predict penumbra, but it is not a reliable way to differentiate tissue that is mildly hypoperfused but not in danger of infarction from tissue that is critically hypoperfused and will subsequently die quickly if not reperfused^29^. According to our results, the perfusion-diffusion mismatch corresponded more to regions with oligaemia than to those with penumbra. The ROIs we put in the normal white matter corresponded to the regions without histological changes but with reduced CBF, which were regions with oligaemia. In this study, for the preservation of morphology, the necessary OEF was approximately 0.4 during the episode. As developed through animal experiments, the concept of penumbra is defined as a dynamic process depending on residual perfusion and duration, with conversion into irreversible neuronal damage over time, progressing from the centre of dense ischaemia to the surrounding tissue with less severe but still critically hypoperfused adjacent areas^30^. Therefore, the penumbra as defined in the present study is different from the conventional definition; we defined it as a compartment corresponding to those with a low risk for morphological damage at the end time point of the experiment. As the duration of ischaemia is prolonged, there might be more progression of the penumbra into the infarction.

Because the variability of blood flow after an attack has a considerable effect on the outcome, a permanent ICA occlusion model was chosen in this study instead of a reversible artery occlusion model. The ligation stroke model is not sufficient to induce severe cerebral ischaemia. Therefore, blockade of blood flow was achieved by intraluminal embolisation with comminuted sutures in our study. Using a middle cerebral artery occlusion model with autologous clots, Liu *et al*.^31^ have reported that infarction can be detected using DWI as early as 1.2 hrs, and lacunar infarcts can be caused by the emboli. However, a limited supply of the cerebral blood flow in the anterior circulation restricts the amount of blood that can be obtained in the superior sagittal sinuses for precise gas analysis. Thus, the correlation of blood oxygen saturation with MR-measured OEF was not performed in our study. Another limitation in our study is that the size of the lesions was not compared with that on TTC slices. On the OEF maps, there were some regions with inappropriate signal curves because of artefacts from the bone-gas interface or from the presence of microbleeding. Compared with the previously MEGESE sequence, the ASE EPI sequence had the advantage of reducing the total scan time, increasing the accuracy associated with the T_2_ calculations and other parameters, and minimising the motion artefacts. However, ghosting artefacts caused by the bone-gas junction persisted. As a result, suitable samples for measurement after the onset of cerebral ischaemia were lessened. Similarly, false-positive regions with an increased OEF were noted in the normal parenchyma on baseline OEF maps, which resulted from artefacts and incorrect estimation. Quantitative measurements of the OEF are currently biased not only by inherent difficulties in T_2_ and CBV quantification but also by inadequacies of the underlying model^32^. The sequence should be further improved for clinical use.

## Conclusion

Using the canine stroke model with catheter-assisted embolisation, we identified the threshold of the OEF to discriminate between the penumbra and infarct on MR OEF imaging through direct comparison with the pathological specimen. After the onset of cerebral ischaemia, cortices with a more elevated OEF tend to progress into infarction more often than those with a mild increase in OEF. The validation of the MR OEF measurement by using ASE might aid in the assessment of oxygen metabolism during the stroke events.

## Additional Information

**How to cite this article**: Chang, F.-Y. *et al*. Determination of oxygen extraction fraction using magnetic resonance imaging in canine models with internal carotid artery occlusion. *Sci. Rep.* **6**, 30332; doi: 10.1038/srep30332 (2016).

## Figures and Tables

**Table 1:** Mean OEF values of the core, penumbra, and normal white matter at different times and 95% confidence intervals (CIs).

Time	Core (95% CI lower, upper)	Penumbra (95% CI lower, upper)	Normal White Matter (95% CI lower, upper)
30 min	0.551(0.530, 0.572)	0.391(0.370, 0.412)	0.282(0.261, 0.303)
60 min	0.588(0.569, 0.607)	0.398(0.379, 0.416)	0.284(0.265, 0.303)
90 min	0.597(0.579, 0.614)	0.395(0.378, 0.413)	0.285(0.268, 0.303)
120 min	0.559(0.537, 0.582)	0.399(0.379, 0.420)	0.287(0.266, 0.307)

**Table 2:** Changes of OEF after occlusion in ROIs (x ± s).

	0.5 hr	1 hr	1.5 hr	2 hrs
Core	0.26 ± 0.10	0.30 ± 0.09	0.30 ± 0.09	0.27 ± 0.07
Penumbra	0.10 ± 0.07	0.10 ± 0.07	0.10 ± 0.07	0.10 ± 0.06
Normal White Matter	0.01 ± 0.03	0.01 ± 0.03	0.01 ± 0.03	0.00 ± 0.03
*χ*^*2*^	84.21	94.24	97.03	95.92
*P*	0.00	0.00	0.00	0.00

**Table 3:** Performance of the OEF in the discrimination between the penumbra and core at different times.

Time	Area under ROC	Cutoff value	Sensitivity	Specificity
30 min	0.902	0.49	82.5%	95.0%
60 min	0.956	0.49	92.5%	92.5%
90 min	0.968	0.48	97.5%	92.5%
120 min	0.969	0.45	95.0%	90.0%
